# Spatial Distribution Patterns of Dominant Tree Species and Their Associations with Soil Factors in Subalpine Secondary Forests of Western Sichuan

**DOI:** 10.3390/plants14223424

**Published:** 2025-11-08

**Authors:** Jingdong Zhao, Xin Liu, Le Wang, Qiuhong Feng, Chang Gou, Jianhua Bai, Xiaohui Yang

**Affiliations:** 1 Institute of Ecological Conservation and Restoration, Chinese Academy of Forestry, Beijing 100091, China; 2 Key Laboratory of Ecological Restoration and Conservation for Forest and Wetland of Sichuan Province, Sichuan Academy of Forestry, Chengdu 610081, China; 3 Sichuan Wolong Forest Ecosystem Research Station, Wolong Forest Ecology Observation and Research Station of Sichuan Province, Wenchuan 623006, China; 4 College of Biology and Food Engineering, Chongqing Three Gorges University, Chongqing 404100, China

**Keywords:** western Sichuan, secondary forest, spatial pattern, *Betula albosinensis*, *Abies fargesii* var. *faxoniana*

## Abstract

Spatial pattern analysis is essential for understanding forest structure and successional dynamics. Focusing on natural secondary forests in the subalpine region of western Sichuan, China, we established two 1-hectare permanent plots to investigate the spatial distribution of dominant tree species and assess the soil’s water-holding properties, aiming to clarify the relationship between species spatial patterns and edaphic conditions. The pioneer species *Betula albosinensis* exhibited a unimodal diameter distribution with scarce seedling presence, indicating limited regeneration. In contrast, *Abies fargesii* var. *faxoniana* showed a typical inverse J-shaped diameter distribution, suggesting stable population recruitment. At fine spatial scales, dominant species generally exhibited aggregated distributions, with *A. fargesii* var. *faxoniana* seedlings showing the strongest clumping; however, as the spatial scale increased, distributions tended toward randomness, likely due to self-thinning and density-dependent interactions. Bivariate spatial association analysis revealed that *B. albosinensis* was positively associated with *A. fargesii* var. *faxoniana* and *Picea asperata* at small scales, suggesting a potential facilitative effect of *B. albosinensis* on Pinaceae species. Moreover, capillary water-holding capacity was significantly higher in areas with greater conifer dominance, underscoring the strong environmental filtering effect of microhabitat moisture on community spatial structure. Collectively, our results suggest an ongoing mid- to late-successional shift from pioneer broadleaved to shade-tolerant conifer dominance, with concurrent changes in species composition and soil conditions. This study provides empirical insight into spatial successional processes and highlights their ecological implications for hydrological regulation in subalpine secondary forests.

## 1. Introduction

Forest ecosystems play a fundamental and irreplaceable role in sustaining global ecological stability, performing critical functions such as water regulation, carbon sequestration, oxygen production, and biodiversity conservation [1]. Among the structural attributes of forest communities, the spatial distribution patterns of trees and their interspecific interactions are recognized as key determinants of community stability and species coexistence mechanisms [2]. Such patterns are commonly examined using multiscale, spatially explicit methods—particularly point pattern analysis—that quantitatively characterize species distribution and interspecific interactions. Such methods have proven indispensable for elucidating the processes underlying community assembly and successional trajectories in forest ecosystems [3]. For example, Zhang et al. (2022), in a study of temperate mixed conifer–broadleaf forests in the Changbai Mountains of China, reported that dominant tree species exhibited pronounced clustering across spatial scales in both disturbed (e.g., selectively logged) and undisturbed plots [4]. Similarly, Nguyen et al. (2016) demonstrated that within a 15 m scale in tropical broad-leaved forests of north-central Vietnam, 12.4% of tree species pairs displayed significant spatial associations, predominantly characterized by positive co-occurrence, suggesting the prevalence of facilitative interactions in that community context [5]. Collectively, these findings underscore the value of multiscale spatial analysis in capturing the complexity of tree–tree interactions. Despite these advances, systematic studies on the spatial distribution and interspecific associations of dominant tree species remain limited in the high-elevation forests of Sichuan and Tibet—regions characterized by natural secondary forests shaped by intensive historical logging and now in active phases of natural regeneration [6].

The interplay between forest spatial structure and soil environmental heterogeneity represents a central mechanism influencing community assembly and successional dynamics [7]. According to niche-based theory, spatial heterogeneity in key edaphic factors—especially soil moisture and nutrient availability—acts as a resource filter influencing species regeneration, competitive dynamics, and spatial distribution within forest stands [8]. Among these, soil moisture functions as a critical limiting factor, especially in water-stressed environments, where its spatial heterogeneity governs the microsite suitability for seed germination, seedling establishment, and sapling survival. As a result, individual trees frequently exhibit aggregated distributions within moisture-favorable microsites [9]. Concurrently, interspecific differences in water-use strategies and drought resistance traits can mediate species-specific responses to soil moisture gradients, contributing to spatial complementarity or niche partitioning among coexisting species [10]. In turn, vegetation can modify soil properties through feedback mechanisms, including canopy interception of precipitation, litter input and decomposition, and belowground root activity, thereby influencing soil water-holding capacity and nutrient cycling processes [11]. Despite the theoretical significance of these interactions, empirical evidence remains limited regarding how dominant tree species in disturbed and rapidly regenerating subalpine natural secondary forests respond spatially to local soil moisture gradients. Notably, rigorous spatial assessments remain scarce for high-elevation forests in regions such as western Sichuan and the eastern Tibetan Plateau.

Building upon the above context, this study investigates the spatial distribution patterns of dominant tree species and their associations with soil water-holding capacity in representative subalpine natural secondary forests of western Sichuan. The primary objective is to clarify the ecological drivers of community succession and assess their implications for hydrological processes in these montane ecosystems. Specifically, we advance two working hypotheses: (1) the spatial distribution patterns of dominant species exhibit pronounced scale dependence, characterized by aggregation at finer spatial scales and transitioning toward random or regular distributions with increasing scale; and (2) as typical late-successional conifers, *Abies fargesii* var. *faxoniana* and *Picea asperata* are positively associated with soil water-holding capacity, reflecting the pivotal role of soil moisture as a limiting factor shaping forest regeneration trajectories in high-elevation environments. By integrating spatial point pattern analysis with edaphic measurements, this study aims to provide empirical insights into the mechanisms linking forest structure and soil water availability. The findings are expected to offer a scientific basis for guiding ecological restoration and informing sustainable management strategies for subalpine natural secondary forests undergoing recovery in western Sichuan.

## 2. Results

### 2.1. Diameter Class Structures of Dominant Tree Species

**Figure 1:**
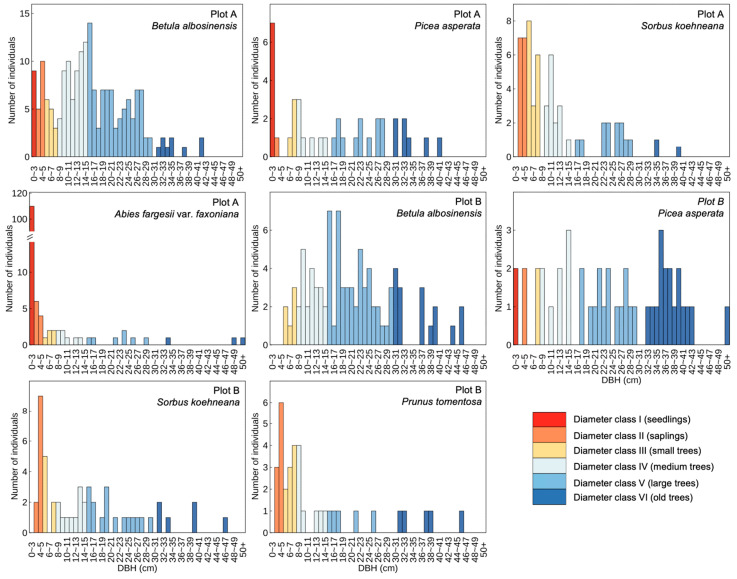
Diameter class structures of dominant tree species in subalpine secondary forests of western Sichuan, China.

In the subalpine natural secondary forests of western Sichuan, the diameter class structures of dominant tree species displayed marked interspecific variation (Figure 1). In Plots A and B, a total of 193 and 90 individuals of *Betula albosinensis* were recorded, respectively. The proportion of seedlings was notably low, and the diameter distribution exhibited a unimodal pattern, with individuals in the medium and large diameter classes comprising the majority. Notably, the distribution in Plot B was more strongly right-skewed than in Plot A, suggesting a declining population structure characterized by recruitment limitation and aging cohorts. In contrast, *A. fargesii* var. *faxoniana* in Plot A exhibited a markedly different pattern, with a total of 142 individuals, of which seedlings accounted for 77%. The diameter distribution followed a typical inverse “J”-shaped curve, indicative of a self-replacing and expanding population with robust natural regeneration dynamics. The two plots also contained 38 and 46 individuals of *P. asperata*, respectively, with a dominance of large and old-aged individuals, reflecting a regeneration bottleneck and weaker population renewal compared to *A. fargesii* var. *faxoniana*. As subordinate tree species within the community, *Sorbus koehneana* and *Prunus tomentosa*—both small-sized broadleaf taxa—were overwhelmingly represented by individuals in the small-diameter classes, further highlighting their suppressed competitive status and understory-adapted life strategies.

### 2.2. Spatial Distribution Patterns of Dominant Tree Species

**Figure 2:**
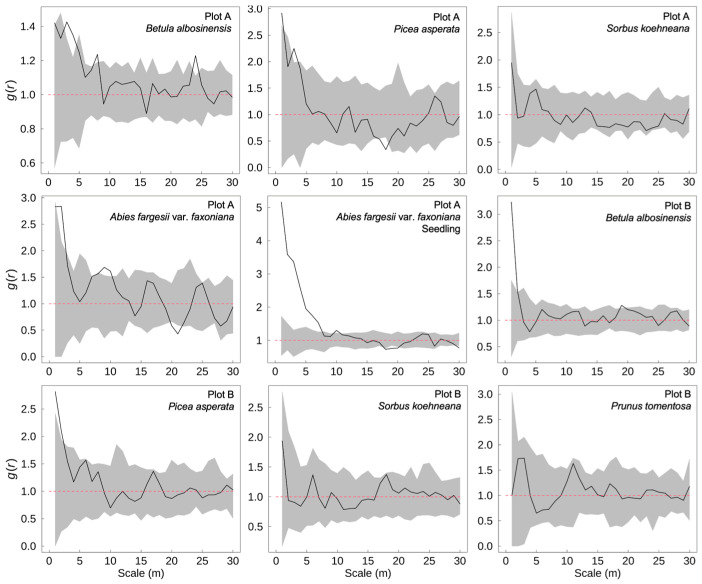
Spatial distribution patterns of dominant tree species in subalpine secondary forests of western Sichuan, China. The black line represents the univariate pair-correlation function *g*(*r*), indicating the degree of spatial aggregation or regularity of a single species at different spatial scales. The gray shaded area shows the 95% confidence envelope generated from 99 Monte Carlo simulations under the null model of complete spatial randomness. Values of *g*(*r*) above the upper envelope indicate significant aggregation, values below the lower envelope indicate spatial regularity, and values within the envelope suggest random distribution.

In the subalpine natural secondary forests of western Sichuan, the spatial distribution patterns of dominant tree species exhibited pronounced scale-dependent characteristics (Figure 2). *B. albosinensis* demonstrated significant aggregation at fine spatial scales in both plots, as well as at broader scales, indicating multi-scale clumping likely associated with seed dispersal limitations and microsite heterogeneity. *P. asperata* also showed significant clustering at small scales, but its distribution became more uniform at larger distances, suggesting increasing intraspecific competition and canopy-level spatial repulsion with increasing scale. *A. fargesii* var. *faxoniana* exhibited strong small-scale aggregation in Plot A, yet shifted toward a uniform pattern at broader scales, reflecting a potential transition from initial recruitment clustering to spatial thinning during stand development. Its seedling cohort showed even more pronounced small-scale aggregation, likely driven by limited seed dispersal and favorable regeneration microsites, and a similar trend toward uniformity at larger scales, possibly due to self-thinning processes or spatial competition during early establishment. By contrast, *S. koehneana* and *P. tomentosa* displayed random spatial distributions across all scales examined, suggesting a more stochastic establishment pattern and a weaker spatial signal likely associated with their subordinate position in the canopy layer.

### 2.3. Interspecific Spatial Associations Among Dominant Tree Species

**Figure 3:**
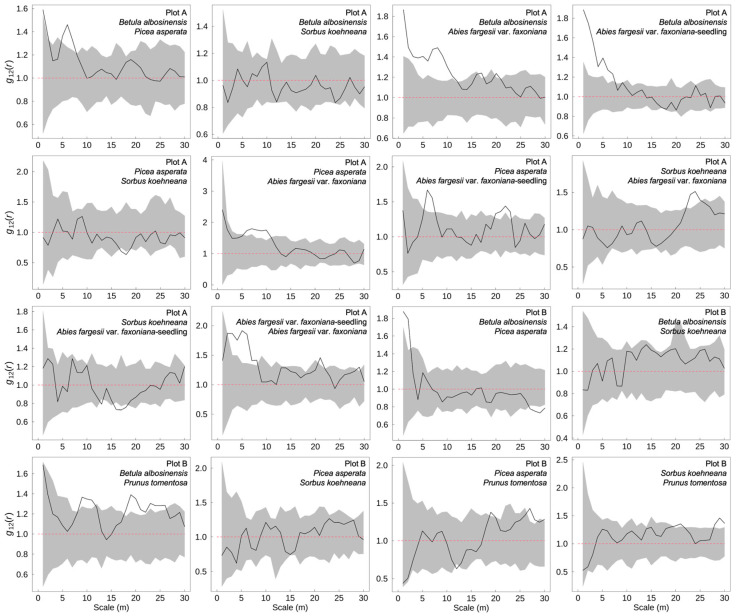
Interspecific spatial associations among dominant tree species in subalpine secondary forests of western Sichuan, China. The black line represents the observed bivariate pair-correlation function *g*_12_(*r*), which describes the spatial association between two species across different spatial scales. The gray shaded area shows the 95% confidence envelope generated from 99 Monte Carlo simulations under the null model of complete spatial randomness. Values of *g*_12_(*r*) above the upper envelope indicate significant positive association, values below the lower envelope indicate negative association, and values within the envelope indicate no significant spatial association between species.

In the subalpine natural secondary forests of western Sichuan, spatial associations among dominant tree species exhibited marked scale dependence (Figure 3). At fine spatial scales, *B. albosinensis* showed significant positive associations with *P. asperata*, *A. fargesii* var. *faxoniana*, and its seedlings, suggesting a potential “nurse effect” during early regeneration stages. However, at broader spatial scales in Plot B, *B. albosinensis* was negatively associated with *P. asperata*, indicating interspecific competition at larger neighborhood extents. *P. asperata* maintained stable and positive spatial associations with *A. fargesii* var. *faxoniana* and its seedlings across multiple scales, which may reflect strong spatial co-occurrence or facilitation among Pinaceae species. In contrast, *S. koehneana* and *P. tomentosa* exhibited weak spatial associations with other dominant taxa, resulting in relatively independent and dispersed distribution patterns overall.

### 2.4. Associations Between Dominance of Dominant Tree Species and Soil Water-Holding Capacities

**Figure 4:**
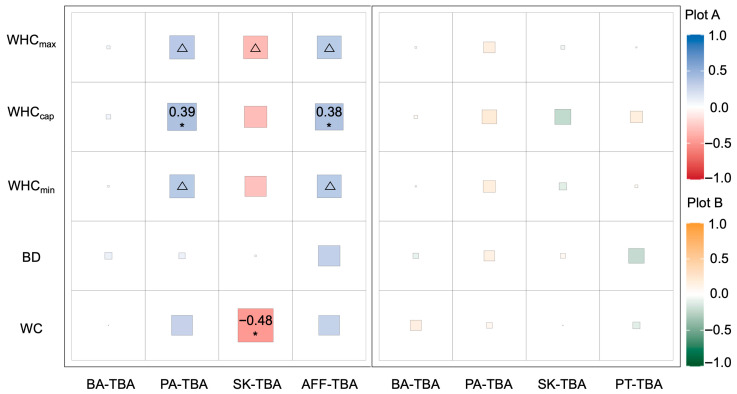
Relationships between the dominance of dominant tree species and soil water-holding capacities in subalpine secondary forests of western Sichuan, China. BD = bulk density (g/cm^3^); WC = gravimetric moisture content (%); WHCmax = maximum water-holding capacity (g/cm^3^); WHCcap = capillary water-holding capacity (g/cm^3^); WHCmin = minimum water-holding capacity (g/cm^3^); TBA = total basal area of each species within the plot (cm^2^). Abbreviations: BA: *Betula albosinensis*; AFF: *Abies fargesii* var. *faxoniana*; PA: *Picea asperata*; SK: *Sorbus koehneana*; PT: *Prunus tomentosa*. *: *p* < 0.05; △: *p* < 0.1. The numerical values in the figure represent Spearman’s rank correlation coefficients (*r*) between tree species dominance and soil water-holding capacity variables.

In Plot A, the dominance of *P. asperata* and *A. fargesii* var. *faxoniana* showed significant positive correlations with soil capillary water content (r = 0.39 and 0.38, respectively; *p* < 0.05) and marginally significant associations with maximum and minimum water-holding capacities (*p* < 0.1), indicating that the distribution and competitive advantage of these conifers may be closely linked to the sustained availability of capillary-bound water in the upper soil layers. Conversely, the dominance of *S. koehneana* exhibited a significant negative correlation with gravimetric soil moisture content (r = −0.48, *p* < 0.05) and a marginally significant association with maximum water-holding capacity (*p* < 0.1), suggesting its occupation of a different soil moisture niche. *B. albosinensis* showed no significant correlations with any soil moisture variables in this plot. In Plot B, no significant relationships were detected between the dominance of any tree species and the measured soil moisture parameters (Figure 4).

## 3. Discussion

### 3.1. Analysis of Diameter Class Structure of Dominant Tree Species

The diameter class structure of tree populations serves as a key diagnostic metric for evaluating stand regeneration dynamics, successional stage, and both ecological and economic value, thereby offering critical insights into population stability and demographic processes [12]. A typical inverse “J”-shaped diameter distribution—marked by a high density of seedlings and small-diameter individuals, followed by a progressive decline in frequency with increasing diameter—is widely regarded as indicative of strong natural regeneration capacity and structural resilience. Such patterns are commonly observed in primary or minimally disturbed forest stands [13]. In the present study, *A. fargesii* var. *faxoniana* in Plot A displayed a clear inverse “J” diameter distribution, suggesting continuous recruitment and sustained population renewal. In contrast, *B. albosinensis* exhibited a unimodal diameter distribution in both plots, characterized by a paucity of seedlings and dominance of intermediate-diameter cohorts. This pattern reflects a substantial decline in regeneration potential, likely signaling population senescence and limited structural continuity [14]. As canopy closure intensifies and surface needle litter accumulates, environmental conditions become increasingly unfavorable for *B. albosinensis* regeneration, further constraining its recruitment niche. A comparable successional dynamic has been documented in the warm-temperate forests of South Africa, where *Podocarpus latifolius* leverages high shade tolerance and early recruitment to maintain persistent regeneration under closed canopies—thus securing a competitive advantage in low-disturbance, low-light environments [15]. Such structural contrasts suggest that the studied subalpine natural secondary forest is currently transitioning through a mid- to late-successional phase, during which light-demanding pioneer species are progressively being replaced by shade-tolerant, late-successional conifers through understory regeneration. This trajectory reflects a gradual shift from a mixed broadleaf–conifer composition toward a conifer-dominated community structure [16]. From a forest management standpoint, if maintaining species diversity and structural heterogeneity is prioritized, targeted interventions—such as gap creation or enrichment planting—may be necessary to facilitate the recruitment of pioneer species. Conversely, aligning with natural successional pathways would support the view that conifer replacement of broadleaf species represents a self-organized and ecologically consistent trajectory [17].

### 3.2. Spatial Distribution Patterns and Associations of Dominant Tree Species and Seedlings

Since the 1950s, extensive logging activities have profoundly altered the structure of alpine primary coniferous forests in western Sichuan, triggering a successional shift toward natural secondary forests dominated by pioneer broadleaf species such as *Betula* spp. [18]. The present study further demonstrates that within these regenerating stands, the spatial distribution of trees exhibits strong scale dependence. At fine spatial scales (<10 m), seedlings of *A. fargesii* var. *faxoniana* display significant clustering, primarily driven by dispersal limitation and microsite heterogeneity. As a gravity- or animal-dispersed species, *A. fargesii* var. *faxoniana* tends to form dense “seed rain” zones in the immediate vicinity of parent trees [19], while localized “safe sites”—including canopy gaps and soil patches with favorable moisture conditions—serve as critical microsites facilitating seedling establishment and promoting spatial aggregation [20,21]. However, as spatial scale increases, overlapping seed shadows and microhabitat averaging diminish local clustering signals, resulting in more randomized spatial patterns at the stand level [22]. As individuals transition into the mature stage, their spatial patterns shift across scales from aggregation to randomness or even regular spacing. This shift reflects the influence of density-dependent processes such as self-thinning and asymmetric resource competition, which gradually regulate stand structure and reduce local clumping [23,24]. In contrast, small-statured or subdominant species such as *S. koehneana* and *P. tomentosa* did not exhibit significant deviations from spatial randomness across the analyzed scales. This may be attributable to their lower population density, broader or more stochastic seed dispersal strategies, and relatively weak environmental filtering, all of which can result in more diffuse and spatially unstructured distribution patterns.

Spatial associations among different tree species and individuals at varying developmental stages exhibit pronounced scale dependence. At fine spatial scales, positive associations typically arise from similar habitat preferences—such as for light, moisture, or nutrients—or from facilitative interactions among neighboring individuals [25,26]. In contrast, negative associations at broader scales are typically driven by density-dependent competition or niche partitioning, which promote spatial segregation among species. In this study, *B. albosinensis*, a pioneer broadleaf species, showed significant positive associations at small scales with both *A. fargesii* var. *faxoniana* seedlings and *P. asperata*, suggesting a potential facilitative role during early succession. These results suggest that *B. albosinensis* may act as a nurse tree, enhancing the microsite conditions beneath its canopy to facilitate the establishment of shade-tolerant conifer seedlings [27,28]. However, as spatial scale increases and trees mature, these initially positive interspecific associations tend to diminish and may shift toward negative correlations. For instance, in Plot B, *B. albosinensis* and *P. asperata* were positively associated at small scales but exhibited significant negative associations at broader spatial extents. This pattern likely reflects increasing spatial exclusion driven by canopy expansion and intensifying competition for below- and aboveground resources [25]. This shift from localized facilitation to broader competitive exclusion exemplifies a key mechanism in secondary forest succession. It reflects the ecological transition from early self-recovery phases toward structurally mature forest communities.

### 3.3. Associations Between Dominant Tree Species and Soil Properties

In subalpine forest ecosystems, soil water availability is widely regarded as a key limiting factor influencing plant community assembly and spatial distribution patterns. This constraint is particularly acute in high-elevation regions, where increased evapotranspiration and marked interannual variability in precipitation amplify water stress [29]. In Plot A, the dominance of *A. fargesii* var. *faxoniana* and *P. asperata* was significantly and positively correlated with soil capillary water-holding capacity, whereas relationships with maximum and minimum water-holding capacities were marginally significant. Although maximum water-holding capacity reflects the total pore volume available for water retention, it includes a considerable fraction of gravitational water, which drains rapidly following saturation and is largely inaccessible to plant roots. In contrast, capillary water is retained in soil micropores through cohesive and adhesive forces between water molecules and mineral particles, constituting the primary source of plant-available water [30]. In this study, capillary water-holding capacity and minimum water-holding capacity represent, respectively, the theoretical maximum water retention of the soil capillary system after gravitational drainage and the stable equilibrium water content under field conditions [31]. These metrics more accurately reflect the actual moisture accessible to roots under natural conditions. Previous research has shown that the fine root systems of *A. fargesii* var. *faxoniana* and *P. asperata* are predominantly concentrated in the upper 0–10 cm of the soil profile, with both fine root biomass and turnover rates declining sharply with depth [32,33]. This shallow rooting distribution, coupled with the lower energetic cost of extracting water from surface layers [34], highlights the strong dependence of these conifers on surface soil moisture. Moreover, tree species adapted to alpine environments often exhibit conservative hydraulic strategies—such as increased organ-specific non-structural carbohydrates concentrations and reduced xylem tracheid diameters—to maintain effective water transport under low-temperature, high-altitude conditions [35]. In this context, the superior capillary water-holding capacity of surface soils provides a reliable and sustained moisture source, enabling conifer species to maintain physiological function and ecological competitiveness under the environmental stresses that are characteristic of high-altitude ecosystems.

**Figure 5:**
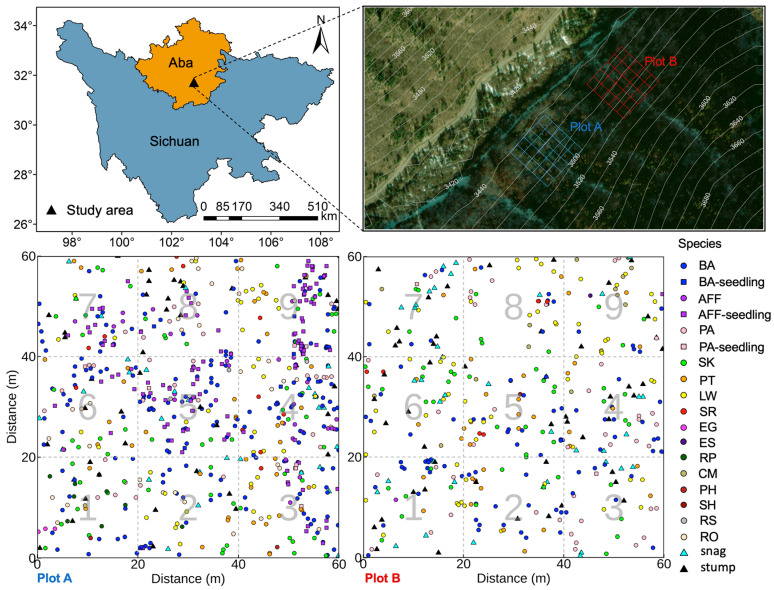
Experimental design and spatial distribution of species within the study plots. Abbreviations: BA: *Betula albosinensis*; AFF: *Abies fargesii* var. *faxoniana*; PA: *Picea asperata*; SK: *Sorbus koehneana*; PT: *Prunus tomentosa*; LW: *Lonicera webbiana*; SR: *Salix rehderiana*; EG: *Eleutherococcus giraldii*; ES: *Eleutherococcus senticosus*; RP: *Ribes pulchellum*; CM: *Cotoneaster multiflorus*; PH: *Prunus hypoxantha*; SH: *Salix hypoleuca*; RS: *Rhododendron simsii*; RO: *Rosa omeiensis*.

Compared to coniferous species, the dominance of *S. koehneana* was significantly and negatively correlated with instantaneous soil moisture content. This pattern may be attributed to its typical occurrence in habitats with reduced canopy closure—such as forest gaps, edges, or shrub-dominated areas—where elevated light availability and intensified evapotranspiration contribute to relatively lower surface soil moisture [36,37]. Spatial association analysis further revealed that *S. koehneana* exhibited significant relationships with other dominant species only at isolated spatial scales, with no consistent associations across the majority of analyzed distances (Figure 3). This spatial segregation likely reflects a differentiated water-use strategy and niche partitioning, which may reduce direct resource competition with co-occurring species and facilitate its persistence and establishment in subalpine communities [38]. Notably, *B. albosinensis* showed no significant correlation with any of the measured soil moisture variables in this study. This suggests that its spatial distribution may be more strongly influenced by other abiotic or biotic factors, such as light availability, disturbance history, or soil nutrient status, rather than by soil water availability alone. This hypothesis requires further validation through targeted investigation. In Plot B, no significant correlations were detected between dominant tree species and measured soil moisture variables, and overall correlation strengths were weaker than those observed in Plot A. This discrepancy may be due to the lower overall tree density (Figure 5), limited seedling recruitment, and relatively reduced intra- and interspecific competition in Plot B, which may diminish the structuring role of soil moisture in shaping spatial distribution patterns [39]. It is important to acknowledge a limitation of this study: all soil moisture data were obtained from the 0–10 cm surface layer. As such, the dataset may not fully capture the vertical heterogeneity of soil water. Given that different species often exhibit distinct vertical root system architectures and water uptake strategies [40], future studies should adopt multi-point and stratified soil sampling across depth profiles, coupled with multi-temporal monitoring techniques. In addition, the current study did not include measurements of soil nutrient concentrations or microbial characteristics, which are also important drivers of species distributions. Future work should integrate these biotic and abiotic factors to provide a more comprehensive understanding of the mechanisms underlying tree spatial patterns.

## 4. Materials and Methods

### 4.1. Study Area

The study area is located in the Miyaluo Forest Region of Lixian County, Aba Tibetan and Qiang Autonomous Prefecture, Sichuan Province, China (31°24′–31°55′ N, 102°35′–103°04′ E), situated on the easternmost fold zone of the eastern Tibetan Plateau. The landscape is characterized by steep mountainous terrain and deeply incised valleys, with pronounced topographic relief. Due to orographic uplift and elevational gradients, the region experiences a cold temperate mountain climate, featuring cold winters and cool summers. Taking Miyaluo Town (elevation ~2760 m) as representative, the area receives an annual precipitation of approximately 700–1000 mm, while the annual evaporation ranges from 1000 to 1900 mm. Mean temperatures in January and July are −8 °C and 12.6 °C, respectively [41].

The forest vegetation in the study area primarily comprises natural secondary forests that have regenerated following intensive logging of primary forests between the 1950s and 1980s [6]. The dominant community type is a mixed conifer–broadleaf forest, exhibiting a well-defined vertical structure and clear species stratification, characteristic of mid- to late-successional stages. The canopy layer is dominated by *B. albosinensis*, *A. fargesii* var. *faxoniana*, and *P. asperata*. The shrub layer is primarily composed of *Lonicera webbiana*, *Rosa omeiensis*, *Cotoneaster multiflorus*, and *Ribes pulchellum*. The herbaceous layer is dominated by *Urtica fissa*, *Primula palmata*, *Galium hoffmeisteri*, and *Luzula plumosa*. According to soil classification, the prevailing soil type is mountain brown forest soil, corresponding to the Cambisols group in the World Reference Base. The soil profile typically has a depth of 40–60 cm. The forest floor (Oh horizon), composed of undecomposed to partially decomposed litter, ranges from 5 to 10 cm in thickness, while the humus layer (Ah horizon) extends approximately 10–20 cm below the surface.

### 4.2. Experimental Design

In early July 2023, two representative 1-hectare plots were established within the core area of the natural secondary forest. Within each plot, a central 60 m × 60 m area was precisely delineated using a total station. This central area was further subdivided into nine standard subplots (each 20 m × 20 m) to facilitate the systematic investigation of stand structure, tree species composition, and spatial distribution patterns. The spatial distributions of trees, shrubs, snags, and stumps within the plots are shown in Figure 5.

A contiguous quadrat method was employed to conduct a systematic survey of the tree and shrub layers within each 20 m × 20 m standard subplot. For all woody individuals with a diameter at breast height (DBH) ≥ 3 cm, we recorded species identity, density, DBH, tree height, crown diameter, and spatial coordinates. For tree seedlings (DBH < 3 cm), height and spatial position were also documented. Based on the survey data, the relative dominance (*RD_i_*) of each species was calculated for both plots (Table 1). Results indicated that in Plot A, the four most dominant species were *B. albosinensis*, *P. asperata*, *S. koehneana*, and *A. fargesii* var. *faxoniana*, together accounting for 88.34% of total dominance. In Plot B, the corresponding dominant species were *B. albosinensis*, *P. asperata*, *S. koehneana*, and *P. tomentosa*, jointly contributing 92.78% of total dominance. Therefore, subsequent analyses of spatial distribution patterns focused on these four dominant tree species in each plot. The *RD_i_* (%) was calculated using the following formula [42]:

(1) $${RD}_{i}=\frac{{TBA}_{i}}{{TBA}_{t}}\times 100\%$$

where *TBA_i_* represents the total basal area at breast height (cm^2^) of species *i*, and *TBA_t_* denotes the total basal area at breast height (cm^2^) of all woody species within the plot.

### 4.3. Determination of Soil Water-Holding Indices

Soil samples were collected from the 0–10 cm surface layer within each 20 m × 20 m subplot, which was evenly divided into four 10 m × 10 m sub-quadrats. Samples were taken from sub-quadrats 1, 2, and 3 using the cutting ring method, and were analyzed separately to determine soil bulk density and water-holding capacity. First, cutting rings filled with undisturbed soil were soaked in water for 12 h to saturate all capillary and non-capillary pores. The saturated rings were then weighed (first weighing). Next, the rings were placed on dry sand for 2 h, allowing non-capillary water to drain, followed by a second weighing. Subsequently, the rings were left on dry sand for another 24 h, at which point the remaining water represented capillary (retained) water. A third weighing was then conducted. Finally, the soil samples were oven-dried to a constant weight to obtain the dry soil mass. Bulk density, gravimetric moisture content, and water-holding capacity were calculated using the following formulas [31]:

(2) $$BD=\frac{m_{s0}}{V}$$

(3) $$WC=\frac{m_{s}-m_{s0}}{m_{s0}}\times 100\%$$

(4) $${WHC}_{max}=\frac{m_{s1}-m_{s0}}{V}$$

(5) $${WHC}_{cap}=\frac{m_{s2}-m_{s0}}{V}$$

(6) $${WHC}_{min}=\frac{m_{s3}-m_{s0}}{V}$$

where BD is the soil bulk density (g/cm^3^), WC is the gravimetric moisture content (%), and *WHC_max_*, *WHC_cap_*, and *WHC_min_* represent the maximum, capillary, and minimum soil water-holding capacities (g/cm^3^), respectively. *m_s_*, *m*_*s*0_, *m*_*s*1_, *m*_*s*2_, and *m*_*s*3_ refer to the fresh weight of the soil in the cutting ring (g), the oven-dry weight (g), the saturated weight after 12 h of soaking (g), the weight after draining non-capillary water (g), and the weight corresponding to capillary water only (g), respectively. V denotes the volume of the cutting ring (cm^3^).

### 4.4. Data Processing

To analyze the spatial distribution patterns of tree species and their interspecific associations, Monte Carlo simulations (99 iterations) were conducted to generate 95% confidence envelopes [24]. The univariate pair correlation function *g*(*r*) was applied to examine the spatial patterns of dominant tree species in both plots and *A. fargesii* var. *faxoniana* seedlings in Plot A [43]. Interspecific spatial associations were further assessed using the bivariate pair correlation function *g*_12_(*r*). Spearman’s rank correlation coefficient was used to assess the relationships between the dominance of each dominant tree species and soil water-holding capacity. All spatial point pattern analyses were performed in RStudio 2023.09.1+494 using the “spatstat” package. Graphical outputs and data visualizations were generated using the “ggplot2” and “corrplot” packages. Spatial data processing and cartographic visualization were carried out in ArcGIS 10.8.

## 5. Conclusions

Based on field investigations conducted in subalpine natural secondary forests of western Sichuan, this study systematically analyzed the diameter class structures, spatial distribution patterns, interspecific spatial associations, and their relationships with soil water properties for dominant tree species. The results indicate that the pioneer broadleaf species *B. albosinensis* exhibits signs of regeneration failure, with a population structure dominated by intermediate diameter classes and a limited seedling bank. In contrast, the shade-tolerant conifer *A. fargesii* var. *faxoniana* shows a typical inverse “J”-shaped diameter distribution, reflecting continuous seedling recruitment and sustained population renewal. This suggests that the forest community is currently undergoing a mid- to late-successional transition, shifting from broadleaf dominance toward coniferous species. In terms of spatial structure, *A. fargesii* var. *faxoniana* seedlings exhibited strong small-scale aggregation, primarily shaped by dispersal limitation and microsite heterogeneity, whereas the overall spatial distribution of dominant species tended toward randomness at the stand level. Spatial association analyses revealed pronounced scale dependence: at fine spatial scales, positive associations were primarily driven by facilitative interactions and shared preferences for similar microhabitats; at broader scales, intensified competition for resources and spatial exclusion led to neutral or negative associations among species. The soil’s capillary water-holding capacity emerged as a key factor regulating the spatial dominance of *A. fargesii* var. *faxoniana* and *P. asperata*, underscoring their reliance on stable surface soil moisture. In contrast, *S. koehneana* was more frequently associated with relatively drier microsites, highlighting the strong environmental filtering effect of soil moisture on species distribution. These findings highlight the close link between regeneration, spatial structure, and soil water availability, offering empirical insights into forest succession and a foundation for adaptive management to support community stability and ecosystem functioning.

## Figures and Tables

**Table 1:** Composition of the tree and shrub layers in the subalpine secondary forests of western Sichuan.

Plot	Type	Species	Count	Mean DBH (cm)	Total Basal Area (cm^2^)	Mean Height (m)	Mean Crown Area (m^2^)	Relative Dominance (%)
A	Tree	BA	184	16.30	48,052.73	8.33	4.26	53.73
		PA	31	19.82	12,160.08	8.00	2.15	13.60
		SK	60	11.50	9860.26	4.00	2.88	11.02
		AFF	32	13.54	8938.48	6.69	3.68	9.99
		PT	45	10.14	5312.97	3.73	2.86	5.94
		SR	11	10.99	1232.28	3.70	2.63	1.38
		PH	2	4.20	29.59	2.05	2.36	0.03
	Shrub	LW	40	7.72	2449.73	3.13	2.47	2.74
		RO	32	4.54	615.54	2.67	1.83	0.69
		RP	8	7.68	427.86	2.88	2.17	0.48
		RS	3	8.43	169.96	2.43	2.55	0.19
		CM	1	13.93	152.41	4.00	4.71	0.17
		EG	3	3.83	35.41	1.93	0.79	0.04
	Seedling	BA–seedling	9	/	/	2.28	/	/
		AFF–seedling	110	/	/	0.88	/	/
		PA–seedling	7	/	/	1.26	/	/
B	Tree	BA	90	20.56	36,323.57	9.24	6.72	38.85
		PA	44	25.73	28,263.38	9.06	3.69	30.23
		SK	51	15.23	13,950.44	4.42	4.50	14.92
		PT	36	12.91	8211.86	4.10	3.68	8.78
		AFF	1	40.64	1297.05	12.20	12.57	1.39
		SR	6	9.95	574.67	5.17	3.17	0.61
	Shrub	LW	60	7.94	3867.97	3.52	1.74	4.14
		CM	14	6.66	562.39	2.85	3.34	0.60
		SH	1	23.56	435.85	7.50	9.62	0.47
		RP	1	3.80	11.37	3.00	0.79	0.01
		ES	1	3.24	8.24	2.50	0.94	0.01
	Seedling	AFF–seedling	1	/	/	0.80	/	/
		PA–seedling	2	/	/	1.65	/	/

Plot size: 60 × 60 cm^2^. Abbreviations: BA: *Betula albosinensis*; AFF: *Abies fargesii* var. *faxoniana*; PA: *Picea asperata*; SK: *Sorbus koehneana*; PT: *Prunus tomentosa*; LW: *Lonicera webbiana*; SR: *Salix rehderiana*; EG: *Eleutherococcus giraldii*; ES: *Eleutherococcus senticosus*; RP: *Ribes pulchellum*; CM: *Cotoneaster multiflorus*; PH: *Prunus hypoxantha*; SH: *Salix hypoleuca*; RS: *Rhododendron simsii*; RO: *Rosa omeiensis*.

## Data Availability

Data available on request due to restrictions.

## References

[1] Mori A.S., Lertzman K.P., Gustafsson L. (2017). Biodiversity and ecosystem services in forest ecosystems: A research agenda for applied forest ecology. J. Appl. Ecol..

[2] Liu P., Wang W., Bai Z., Guo Z., Ren W., Huang J., Xu Y., Yao J., Ding Y., Zang R. (2020). Competition and facilitation co-regulate the spatial patterns of boreal tree species in Kanas of Xinjiang, northwest China. Forest Ecol. Manag..

[3] Lin Y.C., Chang L.W., Yang K.C., Wang H.H., Sun I.F. (2011). Point patterns of tree distribution determined by habitat heterogeneity and dispersal limitation. Oecologia.

[4] Zhang M., Wang J., Kang X. (2022). Spatial distribution pattern of dominant tree species in different disturbance plots in the Changbai Mountain. Sci. Rep..

[5] Nguyen H.H., Uria-Diez J., Wiegand K. (2016). Spatial distribution and association patterns in a tropical evergreen broad-leaved forest of north-central Vietnam. J. Veg. Sci..

[6] Zhang Y., Liu S., Ma J. (2006). Water-holding capacity of ground covers and soils in alpine and sub-alpine shrubs in western Sichuan, China. Acta Ecol. Sin..

[7] John R., Dalling J.W., Harms K.E., Yavitt J.B., Stallard R.F., Mirabello M., Hubbell S.P., Valencia R., Navarrete H., Vallejo M. (2007). Soil nutrients influence spatial distributions of tropical tree species. Proc. Natl. Acad. Sci. USA.

[8] Xu W., Ci X., Song C., He T., Zhang W., Li Q., Li J. (2016). Soil phosphorus heterogeneity promotes tree species diversity and phylogenetic clustering in a tropical seasonal rainforest. Ecol. Evol..

[9] Pastur G.J.M., Esteban R.S., Cellini J.M., Lencinas M.V., Peri P.L., Neyland M.G. (2014). Survival and growth of *Nothofagus pumilio* seedlings under several microenvironments after variable retention harvesting in southern Patagonian forests. Ann. Forest Sci..

[10] Negret B.S., Pérez F., Markesteijn L., Castillo M.J., Armesto J.J. (2013). Diverging drought-tolerance strategies explain tree species distribution along a fog-dependent moisture gradient in a temperate rain forest. Oecologia.

[11] Yang J., Wang A., Shen L., Dai G., Liu Y., Zhang Y., Fei W., Wu J. (2024). The impact of canopy on nutrient fluxes through rainfall partitioning in a mixed broadleaf and coniferous forest. Forests.

[12] Rubin B.D., Manion P.D., Faber-Langendoen D. (2006). Diameter distributions and structural sustainability in forests. Forest Ecol. Manag..

[13] Stephens S.L., Gill S.J. (2005). Forest structure and mortality in an old-growth Jeffrey pine-mixed conifer forest in north-western Mexico. Forest Ecol. Manag..

[14] Heiri C., Wolf A., Rohrer L., Bugmann H. (2009). Forty years of natural dynamics in Swiss beech forests: Structure, composition, and the influence of former management. Ecol. Appl..

[15] Adie H., Lawes M.J. (2009). Explaining conifer dominance in Afrotemperate forests: Shade tolerance favours *Podocarpus latifolius* over angiosperm species. Forest Ecol. Manag..

[16] Adie H., Lawes M.J. (2009). Role reversal in the stand dynamics of an angiosperm–conifer forest: Colonising angiosperms precede a shade-tolerant conifer in Afrotemperate forest. Forest Ecol. Manag..

[17] Dong L., Jin X., Pukkala T., Li F., Liu Z. (2019). How to manage mixed secondary forest in a sustainable way?. Eur. J. Forest Res..

[18] Ma J., Liu S., Shi Z., Zhang Y., Kang B., Chen B. (2008). Changes in species composition and diversity in the restoration process of sub-alpine dark brown coniferous forests in Western Sichuan Province, China. Front. For. China.

[19] García D., Obeso J.R., Martínez I. (2005). Spatial concordance between seed rain and seedling establishment in bird-dispersed trees: Does scale matter?. J. Ecol..

[20] Barbeito I., Fortin M.J., Montes F., Cañellas I. (2009). Response of pine natural regeneration to small-scale spatial variation in a managed Mediterranean mountain forest. Appl. Veg. Sci..

[21] Zhu J., Zhu C., Lu D., Wang G.G., Zheng X., Cao J., Zhang J. (2021). Regeneration and succession: A 50-year gap dynamic in temperate secondary forests, Northeast China. Forest Ecol. Manag..

[22] Chu G.M., Wang M., Zhang S.X. (2014). Spatial point patters of *Anabasis aphylla* populations in the proluvial fan of south Junggar basin. Sci. Silvae Sin..

[23] Yao J., Zhang X., Zhang C., Zhao X., Von Gadow K. (2016). Effects of density dependence in a temperate forest in northeastern China. Sci. Rep..

[24] Li Y., Li M., Ming A., Wang H., Yu S., Ye S. (2021). Spatial pattern dynamics among co-dominant populations in early secondary forests in Southwest China. J. For. Res..

[25] Martínez I., Wiegand T., González-Taboada F., Obeso J.R. (2010). Spatial associations among tree species in a temperate forest community in North-western Spain. Forest Ecol. Manag..

[26] Liu J., Bai X., Yin Y., Wang W., Li Z., Ma P. (2021). Spatial patterns and associations of tree species at different developmental stages in a montane secondary temperate forest of northeastern China. PeerJ.

[27] Stark H., Nothdurft A., Block J., Bauhus J. (2015). Forest restoration with *Betula* ssp. and *Populus* ssp. nurse crops increases productivity and soil fertility. Forest Ecol. Manag..

[28] Shemesh H. (2025). A systematic review of nurse objects as safe sites for seedling establishment and implications for restoration. New Phytol..

[29] Andrus R.A., Harvey B.J., Rodman K.C., Hart S.J., Veblen T.T. (2018). Moisture availability limits subalpine tree establishment. Ecology.

[30] Gavrilescu M. (2021). Water, soil, and plants interactions in a threatened environment. Water.

[31] Zhang W., Xu B. (1986). Methods of Long-Term Forest Soil Research.

[32] Liu S., Luo D., Yang H.G., Shi Z.M., Liu Q.L., Zhang L., Kang Y., Ma Q. (2018). Fine root biomass, productivity and turnover of *Abies faxoniana* primary forest in sub-alpine region of western Sichuan, China. Chin. J. Ecol..

[33] Liu S., Luo D., Yang H., Shi Z., Liu Q., Zhang L., Kang Y. (2018). Fine root dynamics in three forest types with different origins in a subalpine region of the Eastern Qinghai-Tibetan Plateau. Forests.

[34] Horne D.J., Scotter D.R. (2016). The available water holding capacity of soils under pasture. Agr. Water Manag..

[35] Pan S.A., Hao G., Li X., Feng Q., Liu X., Sun O.J. (2022). Altitudinal variations of hydraulic traits in Faxon fir (*Abies fargesii* var. *faxoniana*): Mechanistic controls and environmental adaptability. For. Ecosyst..

[36] Li D.M., Xu Z.J.R., Ma W.B., Duan Q.Y., Yang Z.X., Bai B., Li T., Yang C.B., Wang Q.Y. (2019). A preliminary report on wild germplasm resources and application suggestions of *Sorbus* in Sichuan. J. Sichuan For. Sci. Technol..

[37] Liu Y., Li Z., Chen Y., Jin L., Li F., Wang X., Long Y., Liu C., Kayumba P.M. (2025). Global greening drives significant soil moisture loss. Commun. Earth Environ..

[38] Balvanera P., Quijas S., Pérez-Jiménez A. (2011). Distribution patterns of tropical dry forest trees along a mesoscale water availability gradient. Biotropica.

[39] Kulha N., Honkaniemi J., Barrere J., Brandl S., Cordonnier T., Korhonen K.T., Kunstler G., Paul C., Reineking B., Peltoniemi M. (2023). Competition—Induced tree mortality across Europe is driven by shade tolerance, proportion of conspecifics and drought. J. Ecol..

[40] Hackmann C.A., Paligi S.S., Mund M., Hölscher D., Leuschner C., Pietig K., Ammer C. (2025). Root water uptake depth in temperate forest trees: Species-specific patterns shaped by neighbourhood and environment. Plant Biol..

[41] Feng Q.H., Huang J.S., Xu Z.J.R., Xie D.J., Liu X.L., Pan H.L., Liu S.R. (2016). Effects of density adjusting on biomass and biodiversity of artificial *Picea asperata* forest in sub-alpine region of western Sichuan, China. J. Sichuan For. Sci. Technol..

[42] Li J.Q., Niu S.K., Liu Y.H. (2017). Forest Ecology.

[43] Ma F., Wang S., Sang W., Zhang S., Ma K. (2024). Spatial distribution and sustainable development of living woody and coarse woody debris in warm-temperate deciduous broadleaved secondary forests in China. Plants.

